# Development and Pharmacokinetic Evaluation of Newly Formulated Letrozole Non-Aqueous Nanoemulgel Transdermal Systems for Hormone-Dependent Breast Cancer Therapy

**DOI:** 10.3390/pharmaceutics17111444

**Published:** 2025-11-08

**Authors:** Husam M. Younes, AlSayed A. Sallam, Loai Ahmad Saifan, Aya M. Ghanem, Enam A. Khalil, Ehab A. Abu-Basha, Ahmad Y. Abuhelwa

**Affiliations:** 1 Tissue Engineering & Nanopharmaceuticals Research Laboratory, Office of the Vice President for Research and Graduate Studies, Qatar University, Doha P.O. Box 2713, Qatar; 2 Biomedical Research Center, QU Health, Qatar University, Doha P.O. Box 2713, Qatar; 3 Al Taqaddom Pharmaceutical Industries, Amman 11196, Jordan; a.sallam@tqpharma.com; 4 Department of Applied Science, Faculty of Aqaba College, Al-Balqa Applied University, Salt 19117, Jordan; aya.ghanem@bau.edu.jo; 5 Department of Pharmaceutics and Pharmaceutical Technology, Faculty of Pharmacy, The University of Jordan, Amman 11196, Jordan; ekayoub@ju.edu.jo; 6 Department of Veterinary Basic Medical Sciences, Faculty of Veterinary Medicine, Jordan University of Science and Technology, Irbid 22110, Jordan; abubasha@just.edu.jo; 7 Department of Pharmacy Practice and Pharmacotherapeutics, College of Pharmacy, University of Sharjah, Sharjah 27272, United Arab Emirates; ahmad.abuhelwa@sharjah.ac.ae

**Keywords:** letrozole, non-aqueous nanoemulgel, transdermal delivery, breast cancer, aromatase inhibitors, pharmacokinetics, nanoemulsion

## Abstract

**Background/Objectives:** Breast cancer remains the most prevalent malignancy among women worldwide, with letrozole (LZ) serving as a critical aromatase inhibitor for hormone receptor–positive cases. However, long-term oral administration of LZ is often associated with systemic adverse effects and poor patient compliance. To overcome these limitations, new non-aqueous nanoemulgels (NEMGs) were developed for transdermal delivery of LZ. **Methods:** The NEMGs were formulated using glyceryl monooleate (GMO), Sepineo P600^®^, Transcutol, propylene glycol, and penetration enhancers propylene glycol laurate (PGL), propylene glycol monocaprylate (PGMC), and Captex^®^. Physicochemical characterization, solubility, stability, and in vitro permeation studies were conducted using Strat-M^®^ membranes, while in vivo pharmacokinetics were evaluated in rat models. **Results:** The optimized GMO/PGMC-based NEMG demonstrated significantly enhanced drug flux, higher permeability coefficients, and shorter lag times compared with other NEMGs and suspension emulgels. In vivo, transdermal application of the GMO/PGMC-based NEMG over an area of 2.55 cm^2^ produced dual plasma absorption peaks, with 57% of the LZ dose absorbed relative to oral administration over 12 days. Shelf-life and accelerated stability assessments confirmed excellent physicochemical stability with negligible crystallization. **Conclusions:** The developed LZ NEMG formulations offer a stable, effective, and patient-friendly transdermal drug delivery platform for breast cancer therapy. This system demonstrates potential to improve patient compliance and reduce systemic toxicity compared to conventional oral administration.

## 1. Introduction

Breast cancer is the most commonly diagnosed cancer and the leading cause of cancer-related mortality among women globally [1]. In 2022, over 2.3 million new cases of breast cancer were reported in women, and 670,000 deaths [2]. The cases are expected to increase by 38 percent globally by 2050, with annual deaths from the disease projected to rise by 68 percent, according to a new report from the International Agency for Research on Cancer [3]. While the incidence is relatively lower in Eastern Europe, South America, Southern Africa, and Western Asia, breast cancer remains the most prevalent cancer in women in these regions.

Aromatase Inhibitors (AIs) serve as an alternative to Tamoxifen (TX) for adjuvant therapy in postmenopausal women with hormone-receptor-positive breast cancer. Available options include Anastrozole and Letrozole (LZ) for five years and Anastrozole and Exemestane following two to three years of TX treatment for a total of five years of hormonal therapy. It is recommended that five years of LZ therapy be considered following an initial five years of TX. Patients on AIs should be regularly monitored for bone density, cardiovascular risk, and treatment outcomes [4].

LZ is rapidly and completely absorbed from the gastrointestinal tract, with food having no significant impact on its absorption. It is slowly metabolized into an inactive metabolite, and its glucuronide conjugate is primarily excreted via renal pathways, representing the primary clearance method. Approximately 90% of radiolabeled LZ is excreted in the urine. The terminal elimination half-life of LZ is approximately two days, and steady-state plasma concentrations are reached within 2–6 weeks of daily 2.5 mg dosing. At steady state, plasma levels are 1.5–2 times higher than predicted from a single dose, showing mild non-linearity in LZ pharmacokinetics when administered daily. These steady-state levels remain consistent over prolonged periods, although continuous accumulation does not occur. LZ is weakly bound to plasma proteins and has a large volume of distribution, approximately 1.9 L/kg [5,6].

Long-term oral administration of LZ may cause several side effects in women, including hot flashes and sweating (affecting about 30% of women), joint pain (20%), fatigue (20%), mild skin rashes (10%), headaches (10%), dizziness (10%), nausea (10%), fluid retention causing swelling in the ankles or fingers (10%), loss of appetite, indigestion, hair thinning, diarrhea, constipation, vaginal dryness, decreased libido, mood changes, cough, and breathlessness (affecting fewer than 10% of women) [7]. Additionally, there may be a slight increase in cholesterol levels and a reduction in bone density due to the prolonged absence of estrogen, affecting fewer than 5% of women. Fluctuations in LZ plasma levels after oral dosing often intensify side effects [8].

One potential solution to such a challenge with orally administered LZ is its administration utilizing transdermal drug delivery systems (TDDS), which maintain a constant drug level, potentially reducing side effects. Weekly application and easy discontinuation improve adherence, making TDDS an appealing option. LZ is a white to yellowish crystalline powder freely soluble in dichloromethane, slightly soluble in ethanol, and practically insoluble in water (102 mg/L at 25 °C). It has a molecular weight of 285.31 g/mol, an empirical formula of C_17_H_11_N_5_, and a melting range of 184–185 °C. It is categorized as a lipophilic drug with a log K o/w of 2.22 [9]. LZ’s low molecular weight, good lipophilicity, and small daily dose make it an excellent candidate for TDDS. Recent studies have demonstrated that LZ formulated as a drug-in-adhesive transdermal patch offers prolonged activity and reduced dosing frequency, which enhances patient compliance [10,11].

Nano-dispersed systems like liposomes, nanoemulsions, and lipid nanoparticles are increasingly used for controlled release and skin targeting [12]. These nano-drug lipid carriers improve drugs’ physical and chemical stability by preventing water from entering the lipid particle core, enhancing percutaneous absorption, and enabling controlled release and targeting [13,14,15]. Thus, nano-lipid carriers offer strong potential for TDDS by passing through ≤100 nm pores, reducing clearance, and allowing deep, targeted, and sustained drug deposition [16,17].

Individually, the components of nanoemulsions can enhance transdermal drug delivery, and their combination creates a synergistic effect that significantly improves drug flux across the skin and bioavailability [18,19,20]. Also, from a pharmaceutical technology perspective, nano-dispersed DDS offers a particulate system that can be produced using established high-pressure homogenization techniques, enabling industrial-scale production [12]. Research also indicates that integrating a nano-dispersed system into a hydrogel can result in a thickened matrix formula for TDDS, further advancing this delivery method [21,22,23].

Non-aqueous nanoemulgels are currently emerging as a promising class of nanocarriers for transdermal drug delivery because they integrate the high solubilization potential of nanoemulsions with the spreadability and bioadhesion of gels, while avoiding water-related instabilities such as hydrolysis and microbial growth [24]. These systems are particularly advantageous for moisture-sensitive or lipophilic active pharmaceutical ingredients, offering enhanced chemical stability, prolonged residence time, and improved skin permeation [11,25]. Recent studies show these systems can include permeation enhancers, optimize droplet size, and provide controlled release with higher skin flux than aqueous formulations [15,26]. Moreover, patented designs and proof-of-concept applications, including non-aqueous nanoemulgels for transdermal delivery of potent small molecules and localized therapy, underscore their translational potential as patient-friendly, non-invasive drug delivery platforms [15,18].

This research focuses on developing and in vivo testing newly formulated non-aqueous nanoemulgels designed for topical and transdermal delivery of the potent aromatase inhibitor LZ. The drug flux optimization was achieved by incorporating nanoemulsions and penetration enhancers into TDDS while exploring the synergistic effects of this combination.

## 2. Materials and Methods

### 2.1. Materials

Letrozole powder (purity 99.80%) was purchased from Jiangsu Sainty Handsome Co., Ltd. (Nanjing, China). Methanol and Acetonitrile (HPLC grades), sodium hydroxide (NaOH), hydrochloric acid (HCl), and hydrogen peroxide (H_2_O_2_) were purchased from Merck (Darmstadt, Germany). Ultra-pure water was obtained from the Milli-Q^®^ system (Molsheim, France). All chemicals were of an analytical grade and used as received. Transcutol^®^ (diethylene glycol monoethyl ether), Labrasol^®^ (Caprylocaproyl polyoxyl-8 glycerides), Propylene glycol monocaprylate (PGMC), Propylene glycol laurate (PGL), and Peceol^®^ (Glyceryl monooleate of 40% content) were provided by Gattefossé Co. (Saint-Priest, France). Sepineo P 600^®^ supplied by Seppic Sciences (La Garenne-Colombes, France). According to the supplier’s brochure, Sepineo^®^ P 600 is a ready-to-use, concentrated dispersion that consists of acrylamide/sodium acryloyldimethyl taurate copolymer, isohexadecane, and polysorbate 80. Propylene Glycol USP/EP was obtained from Dow Chemical Company (Midland, MI, USA). Captex^®^ 170 EP (Coco-Caprylate/Caprate) and Campul^®^ (GMO of 60% content) purchased from Abitec Corporation (Columbus, OH, USA). Myverol^®^ (GMO of 90% content) was provided by Univar Solutions (Downers Grove, IL, USA). Ethyl acetate was purchased from Tedia Company (Fairfield, CT, USA). Potassium dihydrogen phosphate and Tadalafil powder were purchased from Sigma-Aldrich GmbH (Taufkirchen, Germany).

### 2.2. Methods

#### Preparation of Non-Aqueous Nanoemulgels and Suspension Emulgels

All nanoemulgels developed in this study were formulated without the inclusion of water; therefore, they were designated as non-aqueous nanoemulgels. Nonetheless, they are generally referred to simply as nanoemulgels. The following represents the general procedure followed in nanoemulgel preparation. A lipid-containing phase was prepared by dispersing Sepineo P600^®^ (SG) into glycerol monooleate^®^ (GMO) with continuous stirring in a water bath maintained at 50 °C until a clear and uniform mixture was obtained. Separately, a drug solution was formulated by dissolving LZ in Transcutol^®^ (TC) under gentle stirring in a water bath at 70 °C. Once LZ was dissolved entirely, propylene glycol (PG) was added gradually, dropwise, with continuous mixing until a transparent solution was formed. The (LZ–TC–PG) solution and the (GMO–SG) mixture were equilibrated in a water bath at 70 °C. The drug solution was slowly incorporated into the lipid-containing mixture with gentle stirring until a homogeneous nanoemulgel was obtained. The gelling behaviour of the mixtures was due to the acrylamide/sodium acryloyldimethyl taurate copolymer.

A nanoemulgel containing 1.5% *w*/*w* LZ was initially prepared for the emulgel suspension preparation. Subsequently, 95 g of this nanoemulgel was mixed thoroughly with 5 g of finely powdered LZ (passed through a 250 µm mesh) until a visually uniform suspension emulgel was achieved. The penetration enhancer (PE), either PGL, PGMC, or Captex^®^, was incorporated by adding it to the hot (GMO-SG) mixture before emulsification. Initial formulation trials employed different grades of GMO to optimize nanoemulgel formation. For permeation studies, the selected GMO was Abitec/Campul^©^ GMO, which contained 60% glyceryl monooleate.

### 2.3. In Vitro Studies

#### 2.3.1. Chromatographic Analysis

An Acquity Waters Ultra Performance Liquid Chromatographic (UPLC) system with a UV-VIS detector, auto-sampler, and column oven was employed for the LZ in vitro analysis. Chromatographic separation was achieved using an Acquity UPLC BEH C-18 column (2.1 × 50 mm, 1.7 µm particle size) maintained at 25 °C. Isocratic elution was performed using acetonitrile: water (35:65, *v*/*v*). The flow rate was set at 0.3 mL/min, and the UV-detection was carried out at λ = 238 nm. Sample injection volume was set to 1 µL, and the total run time was 5 min, while the retention time of LZ was 1.8 min. A 1000 μg/mL stock solution of LZ was prepared by accurately weighing 25 mg of LZ in 25 mL of methanol. Standard solutions of LZ were prepared daily from the stock solution and diluted as needed with methanol. Solutions were filtered through a 0.45 mm membrane filter (PTFE) before injection. The method was validated according to the International Conference on Harmonization (ICH) guidelines for specificity, linearity, range, accuracy, precision, system suitability, detection limit, and robustness [27].

#### 2.3.2. Forced Degradation Studies & Specificity

LZ (100 µg/mL) was subjected to stress conditions, including acidic, basic, oxidative, thermal, and photolytic degradation, to assess the stability-indicating capability of the developed UPLC method. Acidic, basic, and oxidative degradation were performed in 0.1 M HCl, 0.1 M NaOH, and 5% H_2_O_2_. Solutions were refluxed at 90 °C for 30 min using a silicon oil bath equipped with a condenser. Thermal degradation was performed by refluxing the LZ solution at 90 °C for 30 min. Photolytic degradation was carried out by exposing the LZ solution to UV light for 2 h (λ = 365 nm).

#### 2.3.3. Stability Studies

The stability of LZ was evaluated in the presence of various nanoemulgel components. Mixtures prepared for the saturated solubility studies were stored at 50 °C and analyzed using the proposed method at predetermined intervals. To assess assay stability, nanoemulgel samples were kept in sealed flasks at room temperature and 5 °C for 48 h before analysis. To simulate the duration of the diffusion studies, the standard solution of LZ in the receiving medium was maintained on a water bath at 32 °C for a maximum of 21 days and then analyzed using the proposed method. Similarly, stability studies on nanoemulgels were done by keeping samples for 1 month at 50 °C. A shelf-life stability study was performed for almost 2 years on samples of LZ nanoemulgels kept at room temperature, protected from light.

#### 2.3.4. LZ Assay & Recovery

Nanoemulgels were prepared as described using SG, GMO, TC, and PG. 10 g portions of the prepared emulgel were spiked with 0.5, 1, 2.5, and 3 mg of LZ, respectively, and analyzed for % recovery using the developed UPLC method.

#### 2.3.5. Sample Preparation for Assay

To test filter compatibility, 0.45 µm PTFE and Nylon filters were used to filter the samples, and the LZ concentration before and after filtration was assessed. The weight of an emulgel equivalent to 25 mg of LZ was placed in a 50 mL volumetric flask, dissolved by using acetonitrile, sonicated for 30 min in a cold-water bath, and finally filtered by using a double 0.45 µm PTFE filter. After three hours of freezing, the solution was allowed to come to room temperature for fifteen minutes. A PTFE filter was used for re-filtration, and acetonitrile was used to dilute 5.0 mL to 50.0 mL in a volumetric flask. The process of freezing followed by filtration was necessary to remove the fatty components of the emulgel.

#### 2.3.6. Solubility & Stability Studies in the Receiving Medium

LZ solubility was evaluated in various solvents, oils, surfactants, and co-surfactants using a thermostatic shaking water bath at 32 °C or 37 °C. Excess LZ was added to 5 mL of medium and shaken for 72 h. Samples were filtered (0.45 µm PTFE), diluted appropriately, and analyzed by UPLC. All experiments were performed in triplicate. The influence of TC concentration on the solubility of LZ in phosphate buffer (pH 6.8) at 32 °C was also investigated using the same procedure to determine an appropriate diffusion medium. To evaluate the stability of LZ in the receiving medium, a standard solution of LZ was maintained at 32 °C in a water bath for up to 21 days. All experiments were performed in duplicate.

#### 2.3.7. Accelerated Stability LZ Gels

The accelerated stability study was conducted by storing the samples in a stability chamber at 50 °C for 40 days. Samples were collected at the beginning and after 40 days and were subsequently analyzed using the UPLC method. Additionally, the samples were examined microscopically at the beginning and end of the study.

#### 2.3.8. In Vitro Diffusion Studies

Diffusion was evaluated using PermeGear^®^ Franz diffusion cells with 25 mm internal diameter (Hellertown, PA, USA) equipped with Merck Strat-M^®^ membranes (Darmstadt, Germany). One gram of gel was placed in the donor compartment, while the receptor chamber contained 20 mL phosphate buffer (pH 6.8) with 20% *v*/*v* TC to maintain sink conditions. The system was maintained at 32 °C using a circulating water bath and stirred at 500 rpm. At defined intervals, 1 mL samples were withdrawn and replaced with pre-warmed fresh medium. Studies were conducted in quadruplicate (*n* = 4) over 15 days. Drug content was determined by the reported validated UPLC method, and cumulative release was plotted as a function of time.

#### 2.3.9. Permeation Parameters

The cumulative amount (µg/cm^2^) of LZ that diffused through the membrane (Q) was plotted against time (t), similar to what we reported earlier [28]. The following equation was used to determine the steady-state flux (J_ss_):

(1) $$J_{\mathrm{ss}}=\frac{dQ/dt}{A}$$

where (A) is the diffusion area (cm^2^) and (dQ/dt, µg/cm^2^·h) is the slope of the regression line of the profile’s linear section. The following equation was used to obtain the LZ permeability coefficient (P, cm/h):

(2) $$P=\frac{J_{\mathrm{ss}}}{C\;A}$$

where the donor compartment’s LZ concentration is denoted by (C, µg).

The lag time (T_lag_, h) was measured at the point where the steady-state line and time axis intersected. It was observed to be used only for LZ suspensions in a pH 6.8 phosphate buffer. For every formulation, the data yielded a negative value. Therefore, a different model was employed to calculate the lag time using the DD Solver software ver. 2010 [29].

Furthermore, Equation (3) was used to determine the permeability enhancement factor (PEF) between chosen NEMGs:

(3) $$PEF=\frac{LZ\;permeability\;from\;a\;NEMG\;patch}{LZ\;permeability\;from\;a\;suspension\;in\;PBS\;6.8.}$$

#### 2.3.10. Statistical Analysis

Arithmetic mean values with standard deviations (mean ± SD) were presented. The sample size was *n* = 3 for solubility and analytical studies, and *n* = 4 for permeation studies. Statistically significant differences were assessed utilizing a two-tailed Student’s *t*-test. For in vivo studies, the sample size was *n* = 12 for each study, and two-way analysis of variance (ANOVA) was utilized. The significance level for all comparisons was set at *p* < 0.05.

### 2.4. In Vivo Studies

#### 2.4.1. Plasma Samples’ Analysis and Preparation

The analysis method was established and developed according to published literature, but with slight modifications to achieve high sensitivity and selectivity [30,31]. The modification involved incorporating tadalafil (TD) as an internal standard. A Shimadzu HPLC system (Shimadzu, Kyoto, Japan) was utilized with an isocratic pump LC-10AD, autosampler SIL-20A, RF-20A-Axs fluorescence detector, and SCL-10A controller, managed by CLASS VP 6.14 software. A Column HiQ Sil C18HS (150 mm × 4.6 mm, 5 µm) with a mobile phase of acetonitrile: phosphate buffer 10 mM 40:60, respectively, was employed in the analysis. No pH adjustment was required. The mobile phase was pumped at a 1.2 mL/min flow rate. The fluorescence detector was monitored at 230 nm as an excitation wavelength and 295 nm as an emission wavelength, and the injection volume was 25 µL.

Collected plasma samples were initially permitted to thaw at ambient temperature. Subsequently, 80 µL of plasma and 40 µL of TD solution in acetonitrile (2 ng/µL) and 1 mL of ethyl acetate were pipetted into a 2 mL centrifuge tube and vortex mixed for 30 s. The samples were centrifuged at 12,000 rpm for 5 min. The ethyl acetate layer was aspirated into a glass tube (12 × 75 mm) and evaporated to dryness under nitrogen at room temperature. The residue was reconstituted with 250 µL of mobile phase and shaken vigorously by a vortex mixer to form a sample solution. 25 µL of the sample solution was injected into the HPLC. Linearity was achieved by spiking LZ in blank plasma to prepare 1, 5, 10, 50, 100, 500, and 1000 ng/mL. The method was validated according to the International Conference on Harmonization (ICH) guidelines for specificity, linearity, range, accuracy, precision, system suitability, detection limit, and robustness [27].

#### 2.4.2. Pharmacokinetics Studies

Since nanoemulgel and suspension gel formulations containing PGMC exhibited superior in vitro permeation, they were selected for in vivo studies. Male Sprague Dawley rats (200–250 g) were used for the in vivo pharmacokinetics testing and were divided into three groups (*n* = 12 per group).

- Group A: Oral administration of LZ solution (12 doses; 1 mg/mL ethanol).
- Group B: Topical application of PGMC nanoemulgel (equivalent to 12 mg LZ).
- Group C: Topical application of PGMC suspension gel (equivalent to 12 mg LZ).

**Figure 1:**
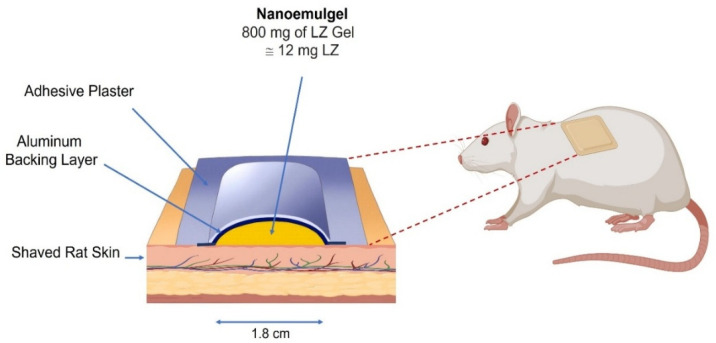
An illustration depicting Nanoemulgel composition and application to a rat’s dorsal back. (Created in BioRender. https://BioRender.com/29pnjso (accessed on 11 October 2025)).

**Figure 2:**
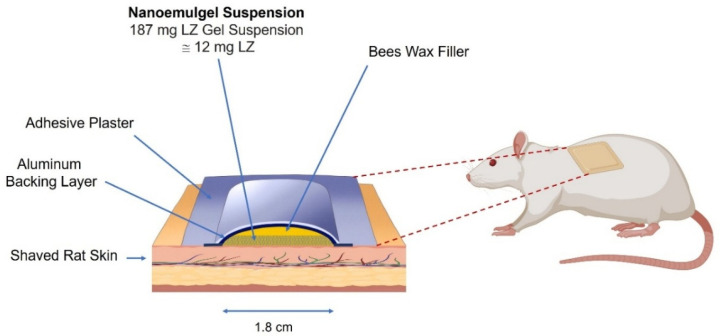
An illustration depicting Gel Suspension’s composition and application to a rat’s dorsal back. (Created in BioRender. https://BioRender.com/rn64fjh (accessed on 11 October 2025)).

For the topical groups B and C, gels were administered as a single dose containing 12 mg of LZ and were left in contact with the rat’s shaved and cleaned dorsal skin for 12 days (Figure 1 and Figure 2). The patches were removed after 12 days. Blood samples were collected from the tail vein at defined intervals for up to 17 days. Plasma was separated (5000 rpm, 5 min) and stored at −20 °C until LZ analysis. All animal studies adhered to the guidelines for animal use established by the ethical board of the Jordan University of Technology, where the study was carried out.

#### 2.4.3. Pharmacokinetics Parameters Assessment

The pharmacokinetic parameters assessed included the area under the plasma concentration–time curve from time zero to the last measurable concentration (AUC_0–t_), the area extrapolated to infinity (AUC_0–∞_), the maximum plasma concentration (C_max_), the elimination half-life (t½), and the time to reach C_max_ (T_max_). These parameters for LZ were calculated using WinNonlin^®^ Version 7.0 (Pharsight Corporation, Mountain View, CA, USA) through noncompartmental analysis. The AUC values were obtained using the linear trapezoidal interpolation method. ANOVA was applied to the log-transformed AUC and C_max_ data, with treatment serving as the main effect. Statistical significance was set at *p* < 0.05. Geometric mean ratios (GMRs) and their 90% confidence intervals (CIs) were calculated for C_max_, AUC_0–t_, and AUC_0–∞_.

## 3. Results & Discussions

### 3.1. UPLC Instrumentation and Conditions

**Figure A1:**
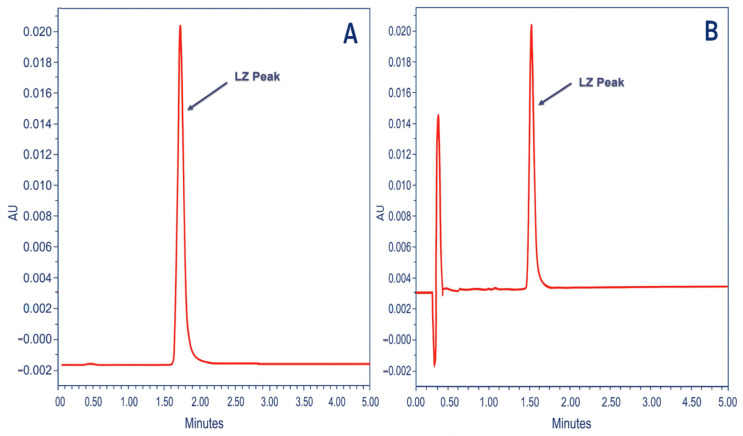
Representative chromatograms of (**A**) LZ 150 µg/mL, (**B**) LZ 0.5 µg/mL.

A mobile phase composed of acetonitrile: water (35:65 *v*/*v*) and a flow rate of 0.3 mL/min was the optimal composition that resulted in a sharp symmetrical LZ peak (Figure A1). The average retention time was 1.8 min with a 0.12% RSD.

### 3.2. Method Validation

System suitability: In all measurements, the peak area showed a variation of less than 2.0% while the average retention time was found to be 1.7 min with an RSD of 0.21%. The capacity and tailing factors were 3 and 1, respectively, as evidence of the method’s suitability for the reported application.

**Figure A2:**
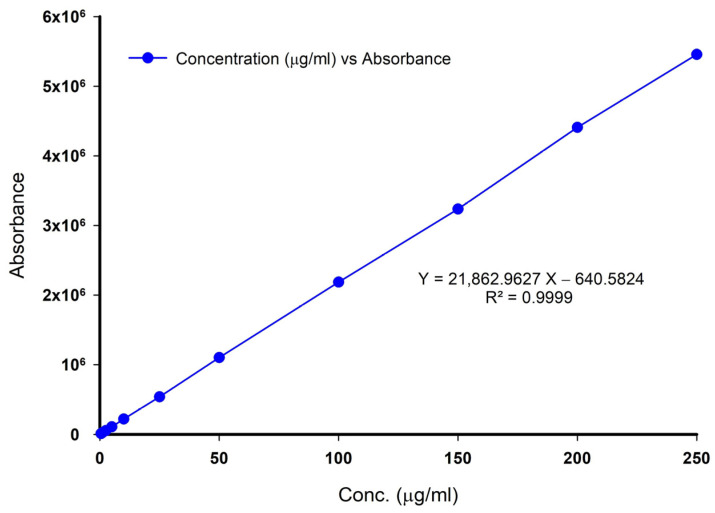
Linearity of LZ.

Linearity, range, and detection limits: The calibration curve for LZ was linear over a concentration range of 2.5–250 µg/mL with a reported correlation coefficient R^2^ equivalent to 0.9999, as shown in Figure A2. Following the ICH guideline, the lowest detectable and quantifiable concentrations of LZ using the assay method were found to be 0.025 µg/mL and 0.125 µg/mL, respectively.

Accuracy & Precision: The percentage recovery showed 102.4, 99.6, 99.8, and 102.0% for spiked amounts of 0.5, 1.0, 2.5, and 3 mg/10 mL nanoemulgel, respectively. Table A1 and Table A2 show the accuracy and precision assessment results after analyzing nanoemulgels. As per the results shown, the method was found to be precise (RSD < 2%) and accurate.

There was no interference between the LZ peak and any of those of the emulsion’s components, which strongly indicates the ability of the reported method to selectively and specifically be used for the analysis of LZ in emulsion formulations containing common oils, surfactants, and co-surfactant mixtures. Similar results were observed for the assay of LZ in nanoemulgels containing various penetration enhancers. As for the possible interference of LZ with PTFE filters, it was found that the percentage recovery of standard aqueous solutions following filtration through PTFE was 99.5% and 99.3%, respectively. The method’s robustness was found acceptable to changes in wavelength, flow rate, and mobile phase composition (%RSD < 2).

### 3.3. Degradation Studies/Specificity

**Figure A3:**
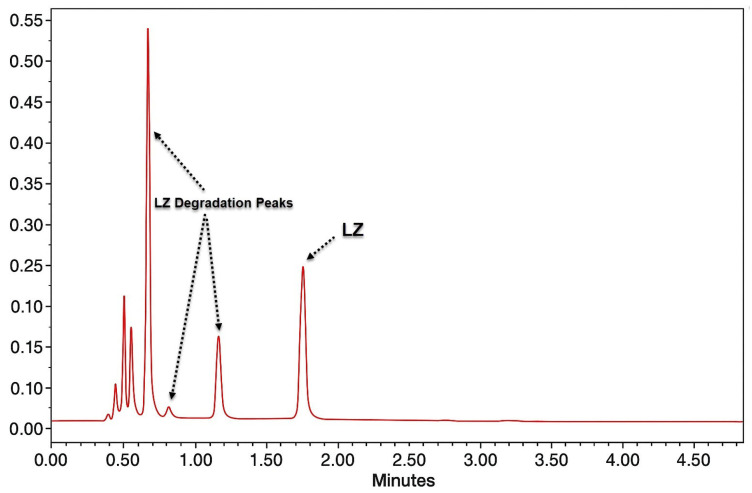
Chromatogram of LZ after treatment with NaOH.

Table A3 reports the percentage of drug recovered/decomposed under the different stress conditions used in the study. LZ was stable under UV-radiation and thermal conditions and showed insignificant instability under acidic and oxidative stress (% decomposed ~ 1.5%). LZ was most sensitive to basic conditions (71.6% decomposition). This instability could be related to the presence of a triazole ring in its structure. In all the analyses, no interference of the LZ peak with any degradation products was observed (Figure A3).

The chromatograms and the amount of LZ recovered after different treatments show that LZ was stable in all other conditions except the alkaline hydrolysis condition. Furthermore, the components of various nanoemulgels did not interfere in the analysis of the standard solution of LZ, nor under forced degradation conditions. The retention time for LZ was 1.8 min for both LZ nanoemulgel samples and standard solutions. The absence of co-eluting degradants and nanoemulgel additives (solvents, surfactants, penetration enhancers, and polymers) was observed with almost purity of 100% for the pure and sample LZ peaks.

### 3.4. Solubility Studies

The solubility of LZ in water, oily solvents, and cosolvents at 32 °C and 37 °C is reported in Table 1. LZ showed a very poor aqueous solubility in water. LZ is a relatively nonpolar molecule that cannot effectively break the lattice structure of water; hence, the aqueous solubility of LZ is low. As shown in Table 1, the solubility of LZ in oily vehicles is much higher than in water. Oily vehicles are essential components of emulsions and emulgels and play a solubilization role for LZ.

The solubility of LZ was found to be the highest in TC and was many times more soluble than in other solvents studied in this investigation. The temperature was also found to affect the solubility of LZ, which increased significantly upon increasing the temperature by 5 °C, as reported in Table 1. The most effective was prominent in the case of TC, which rendered it suitable for use as a cosolvent in formulations of LZ emulgels.

### 3.5. Medium Selection in the Receptor Chamber

**Figure 3:**
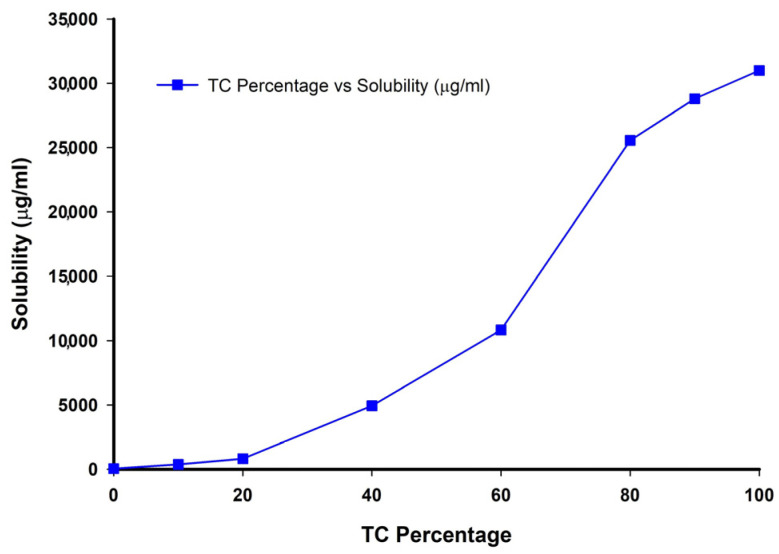
Effect of % TC in phosphate buffer of pH 6.8 on the solubility of LZ (µg/mL) at 32 °C.

The primary criterion for selecting the receptor medium in the diffusion studies was the solubility of LZ in the medium and its capacity to maintain sink conditions for LZ diffusing from the emulgel formulations across the membranes. Therefore, the solubility of LZ in different compositions of phosphate buffer of pH 6.8 (PHB) and TC was tested. It was found that a solution containing 20% TC in PHB is suitable for achieving sink conditions and is also near the physiological condition of the skin. In addition, the medium was replaced every 24 h with fresh solution, a step that kept sink condition fulfilled during the run of the diffusion studies. Figure 3 shows the solubility of LZ in TC/PHB at 32 °C, demonstrating that LZ solubility increases upon increasing the percentage of TC.

### 3.6. LZ Solutions’ Stability in Various Pharmaceutical Additives

The development of LZ nanoemulgels required a comprehensive screening of the solvents and additives used to investigate which have proven to be stable with LZ. Table 2 reports the findings from the stability of LZ solutions in different pharmaceutical additives examined by storing them for a month at 50 °C. It was determined that LZ sample solutions in water, phosphate buffer, alcohols, propylene glycol (PG), Labrasol^®^, and TC had high stability. But for the first time, LZ was discovered to be unstable in PEG 400. PEG’s hydrophilicity and molecular interactions can alter the drug’s solubility and crystal structure, potentially leading to degradation, reduced effectiveness, or even forming a less stable complex [32]. As a result, PG and TC were used in place of PEG 400 as cosolvents in this investigation.

### 3.7. Stability of LZ in the Receiving Medium at 32 °C

The standard LZ solution in the receiving medium remained stable for up to 21 days at 32 °C. Experiments were performed in duplicate, with the mean initial concentration measured at 207.14 µg/mL and 207.22 µg/mL after 21 days, confirming the excellent stability of LZ in the receiving medium.

### 3.8. Stability of Sample Solutions

The stability of sample solutions remained at 48 h in the refrigerator at 5 °C or at room temperature, indicating excellent stability (99.9 ± 1.3% and 99.4 ± 1.7%, respectively). The results confirm that the sample solutions used during the assays were stable up to 48 h at room temperature and in the refrigerator.

### 3.9. Formulation of Nanoemulgels

The main objective of NEMGs formulation is to make drugs more soluble and permeable across biological membranes. The nano-sized droplets in the nanoemulsion boost the drug’s permeation, adding to the synergistic effects of surfactants, co-surfactants, and permeation enhancers. The hydrogel component of NEMGs improves the product’s viscosity, spreadability, and physical stability for TDDS. The cosolvents in the NEMGs also increase the solubility of the drugs. This results in TDDS developing improved pharmacokinetic properties, protecting patients from the systemic side effects commonly associated with oral drug administration. The issue of nanoemulsions being less stable than microemulsions is overcome by including a gelling agent, which produces stable NEMGs [18,23,33].

This study used several non-aqueous components, including oils, surfactants, and polymers [15]. Sepineo P600^®^ is a milky suspension of acrylamide/sodium acryloyldimethyl taurate copolymer that gels on contact with hydrophilic media, containing isohexadecane and polysorbate 80, serving as a surfactant. Glyceryl monooleate (GMO), widely utilized in cosmetics and pharmaceuticals, was included as a cosurfactant. Due to its amphiphilic nature, possessing both hydrophilic and lipophilic domains, GMO functions effectively as an emulsifier and emulsion stabilizer [34]. In addition, GMOs have been reported to enhance transdermal drug delivery by interacting with the stratum corneum, which promotes ceramide extraction and increases lipid fluidity, thereby facilitating drug permeation [35]. Various commercial grades of GMO are available, including Peceol^®^ (40% GMO), Capmul^®^ (60% GMO), and Myverol^®^ (90% GMO). According to suppliers’ information, these products also contain oleic acid di- and triglycerides, along with trace levels of free fatty acids and glycerol. The cosolvents PG and TC were incorporated as the polar, hydrophilic, non-aqueous components of the nanoemulgel system. As previously demonstrated in Table 2, LZ solubility was markedly enhanced in the presence of TC and moderately improved with PG. Notably, both cosolvents have also been reported to act as penetration enhancers [35]. Additional permeation enhancers included propylene glycol laurate (PGL), propylene glycol monocaprylate (PGMC), and Captex^®^ (coco-caprylate/caprate) as summarized in Table 3 [36,37]. It was reported that if the nanoemulsion is between 50 and 200 nm, it appears clear and transparent. However, if it is 500 nm or larger, it will be milky or hazy [18]. This observation was used as a guideline in this investigation. The formation of the nanoemulgel was indicated by the formation of a clear gel.

In the NEMGs matrix, up to 1.5% *w*/*w* LZ may be readily dissolved, as reported in Table 3. Some formulas, such as F08, included 1.52% *w*/*w* LZ in a dissolved state. The solubility of LZ was due to the presence of both PG and TC in a concentration of at least 65% *w*/*w*. While the other GMO grades formed clear gels, NEMG, which included Peceol^®^ in the ratios reported in F07, did not. This result might be explained by the fact that Peceol^®^ included a higher percentage of glyceryl di- and trioleate, which produced a larger molar volume than GMO of Campul^®^ and Myverol^®^ and influenced the size of the emulsion droplets. The formulations without GMO (F08), Campul^®,^ and Myverol^®^ all produced clear gels, which showed the development of nanoemulgels. Transparent gels like F09 and F10 were formed when Campul^®^ and/or Myverol^®^ were mixed with Peceol^®^. The observed texture of the gel indicated that the NEMGs with Myverol^®^ GMO were the stiffest gels, followed by the NEMGs containing Capmul^®^, while the NEMGS with Peceol^®^ exhibited the least stiffness. The LZ components in the NEMGs were found not to influence the structure of the nanoemulgels. The NEMGs produced by Campul^®^ GMO were easier to handle; for this reason, they were selected for further investigation.

The number of NEMGs trials was reduced due to the lengthy runs, which took 15 days, and the large number of samples that needed analysis. For all NEMGS formulations, the PE proportion was maintained at the 5% *w*/*w* level. As reported in Table 4, all penetration enhancers were added to the NEMGs and were found not to affect the gel’s transparency, resulting in a clear, thick gel.

### 3.10. Permeation Studies

In vitro permeation of LZ was conducted at 32 ± 1 °C using a Strat-M^®^ membrane and a phosphate buffer (pH 6.8) containing 20% *v*/*v* Transcutol (TC). The Strat-M^®^ membrane is a multilayer synthetic barrier engineered to mimic the structure and composition of human skin, providing a cost-effective and reliable alternative for in vitro permeation studies. Previous reports have demonstrated a strong correlation between the permeation behavior across Strat-M^®^ and human stratum corneum, confirming its predictive value for percutaneous absorption studies [28].

Uchida et al. reported that the diffusion and partition coefficients of various compounds through Strat-M^®^ closely matched those observed in excised human and rat epidermis [38], supporting its use as a surrogate for biological membranes in permeation testing. Similarly, Haq et al. found comparable diffusion profiles, indicating that Strat-M^®^ is suitable for preliminary in vitro screening of transdermal formulations [39]. In addition, Assaf et al. demonstrated a strong correlation between Strat-M^®^ and human skin in the transdermal evaluation of tamsulosin, further validating its suitability as a substitute membrane [40].

While synthetic membranes can yield results that differ from those of human skin, especially for formulations relying on specific lipid or protein interactions or containing potent penetration enhancers, using the Strat-M^®^ membrane in this study was well justified. The 14-day experimental duration could compromise the integrity of the stratum corneum, leading to its degradation. Therefore, a stable synthetic membrane such as Strat-M^®^ was selected as the most appropriate alternative due to its structural and functional similarity to the human stratum corneum [38,39,40].

**Figure 4:**
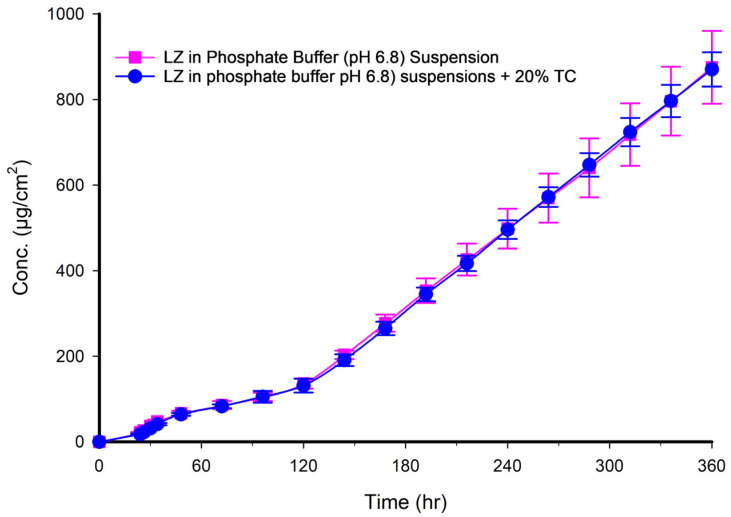
Permeation profile of LZ in phosphate buffer suspensions with and without 20% of TC through Strat^®^ membrane (*n* = 4, mean ± SD).

**Figure 5:**
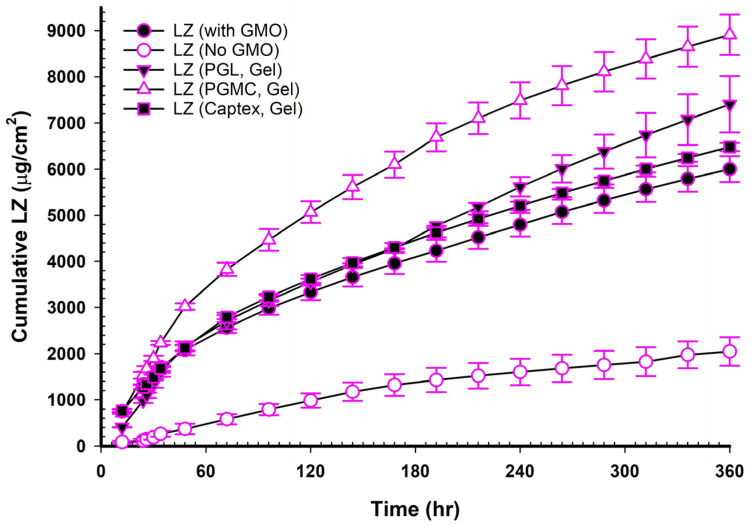
Permeation profiles of LZ in NEMGs containing different PE through the Strat-M^©^ membrane (*n* = 4, mean ± SD).

**Figure 6:**
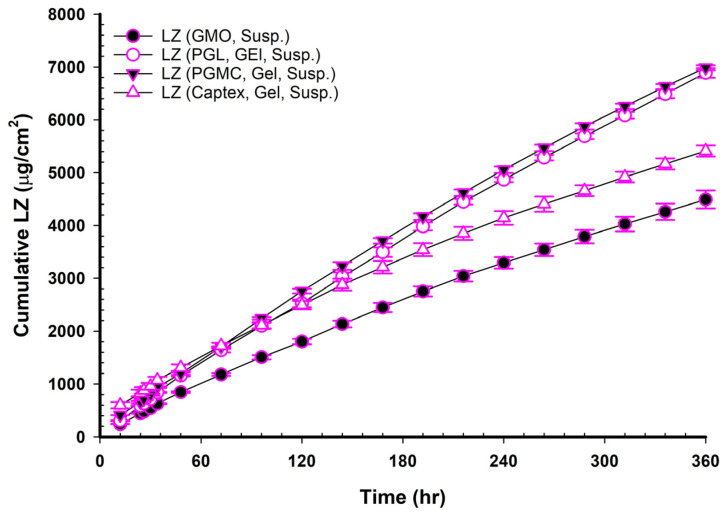
Permeation profile of LZ in emulgel suspensions containing different PE through the Strat^®^ membrane (*n* = 4, mean ± SD).

Figure 4, Figure 5 and Figure 6 show the cumulative amounts of LZ (µg/cm^2^) plotted versus time in hours for LZ suspensions in phosphate buffers with or without 20% *v*/*v* of TC, LZ NEMGs, and LZ gel suspensions. The calculated permeation parameters of LZ from all formulations are summarized in Table 4, Table 5, Table 6 and Table 7.

### 3.11. LZ Permeation from Phosphate Buffer Suspension

The ability of LZ to diffuse through the Strat-M^®^ membrane was examined both with and without 20% *v*/*v* TC suspended in phosphate buffer of pH 6.8. It was observed that LZ had very little permeation, and adding 20% *v*/*v* of TC had no discernible impact on permeation. Nearly identical permeability coefficients were also obtained. Figure 4 and Table 5, respectively, display LZ’s characteristics and in vitro permeation profiles in phosphate buffer with and without 20% *v*/*v* of TC.

### 3.12. LZ Permeation from NEMG Without GMO

In comparison to the LZ suspension in phosphate buffer, it was noted that a greater amount of LZ permeated from NEMG with no GMO. Consequently, the NEMG system facilitated enhanced drug permeation, as indicated by significantly higher flux, increased permeation coefficients, and reduced T_lag_ (*p* < 0.05). This was further evidenced by an enhancement factor that was 16 times greater than that of the LZ suspensions in phosphate buffer, regardless of the presence of TC, as illustrated in Table 6, Table 7 and Table 8. The enhanced permeation observed in the NEMG without GMO can be attributed to the nanostructure of the gel, which provides a significant surface area for membrane contact, thereby facilitating diffusion [41]. Additionally, incorporating PG, TC, and Polysorbate 80 contributes to the enhancement of permeation, which will be elaborated upon in the following sections.

### 3.13. LZ Permeation from NEMG Containing GMO

Figure 5 and Table 6 demonstrate that adding GMO to the NEMGs led to a significant increase in drug permeation and a decreased lag time compared to the NEMG without GMO (*p* < 0.05). Previous studies reported that GMOs interacted with the skin’s stratum corneum to promote drug permeability. GMOs may increase penetration, especially when it comes to transdermal drug delivery [42]. A disruption could aid drug diffusion in the stratum corneum’s lipid composition. Using cosolvents like PG and TC increases penetration when combined with GMOs. Such an effect was well investigated with either cosolvent alone or GMO [42,43,44,45].

### 3.14. LZ Permeation from NEMG Containing GMO and PE

Two main mechanisms explain the synergy between nanoemulsions and penetration enhancers: increased solubility and greater skin contact area, facilitating diffusion. The nano size of the nanoparticles allows them to penetrate skin pores effectively. Meanwhile, penetration enhancers such as surfactants, PG, TC, GMO, PGL, PGMC, and Captex^®^ work in conjunction with the nanoemulsion to disrupt the lipids in the stratum corneum, thereby increasing their fluidity and altering their structure. This disruption leads to voids and disorder within the lipid bilayers, further assisting drug entry into the skin’s deeper layers and systemic circulation [41,44,46,47,48,49,50,51]. PGL, PGMC, and Captex were employed as PEs in various TDDS [40,52,53]. The current investigation highlights the advantages of a nanoemulsion structure, including surfactants, cosolvents, and penetration enhancers, presenting a complex system that merits further exploration. Adding PGL, PGMC, or Captex^®^, combined with GMO, significantly increased LZ permeation (*p* < 0.05). Figure 5 and Table 6 demonstrate how these additional PEs enhanced the permeability of LZ when added to NEMG containing GMO in the following order: GMO/PGMC, NEMG > GMO/PGL, NEMG > GMO/Captex^®^, NEMG > GMO, NEMG > NEMG without GMO. The permeability enhancement followed the following order: GMO/PGMC, NEMG > GMO/PGL, NEMG > GMO/Captex^®^, NEMG > GMO, NEMG > NEMG without GMO, as shown in Table 8. It was evident that the nanoemulsions and penetration enhancers function through distinct mechanisms that may interact to produce a synergistic effect, as already highlighted above. This assertion is backed by the high permeation coefficients and the permeation enhancement factor detailed in Table 6 and Table 8, which align with previously published data [41,46]. The NEMG of GMO/PGMC exhibited a significantly higher permeability coefficient than the others (*p* < 0.05), which led to its selection for in vivo studies, and its gel suspension was also chosen.

### 3.15. Determination of the Permeation Lag Time

The diffusion process in TDDS commences in a non-steady state, illustrated by the initial segment of the curve, while the linear section represents steady-state diffusion. Fick’s second law provides a mathematical framework for describing the non-steady portion of the curve, whereas Fick’s first law offers an expression for the linear segment. The term “lag time” denotes the duration required to achieve a steady state, which can be determined by projecting the linear part of the permeation versus time curve onto the time axis. Due to a high initial penetration level of the LZ, the projection of the linear portion resulted in a negative value for the lag time. Consequently, several models were employed to analyze the diffusion profile segments following the initial segment of the curve and to determine the permeation lag time [54]. The diffusion of LZ in the formulations closely aligned with the Higuchi model with lag time, as indicated by the highest coefficient value (R^2^), which ranged from 0.99 to 0.9999 [29]. The lag times, presented as mean ± standard deviation, for NEMGs and Emulgel suspensions were detailed in Table 6 and Table 7, respectively.

### 3.16. LZ Permeation from Emulgel Suspensions Containing GMO and PE

The LZ permeations from LZ Emulgel suspensions containing GMO and PEs were plotted in Figure 6 and reported in Table 7. The main findings indicated lower permeation rates, longer lag times, and lower PEF values than the corresponding NEMG (Table 8 and Table 9). In contrast to the LZ permeation profiles derived from NEMGs, the permeation profiles of the Emulgel suspension were characterized by a distinct steady state. Permeation enhancement through suspension may involve maintaining a drug concentration in the transdermal vehicle that surpasses its equilibrium solubility. This approach ensured a consistently higher thermodynamic activity, facilitating continuous drug diffusion across the membrane barrier and helping maintain the steady state [51].

The flux of LZ from Emulgel suspensions was lower than that of NEMGs, with a significant difference (*p* < 0.05). The ranking of the permeation coefficients is as follows: GMO/PGMC Emulgel suspension or GMO/PGL Emulgel suspension > GMO/Captex^®^ Emulgel suspension > GMO Emulgel suspension (*p* < 0.05). The reduction in both the flux and the permeation coefficients for the Emulgel suspension was attributed to its behavior as an emulgel rather than a nanoemulgel, which resulted in a loss of the advantages associated with the nanostructure of the emulsion. Because nanoemulgels have a much higher surface area for drug transfer and can more successfully contain penetration enhancers, their drug penetration in TDDS was often better than that of regular emulgels. These factors accelerated and enhanced drug penetration through the skin’s barriers and into the systemic circulation [40]. The presence of nanostructure in the nanoemulgel could not be demonstrated in the suspension forms. However, this advantage was lost after 100 h of the experiment, as will be detailed later. NEMGs had flux comparable to Emulgel suspensions after 100 h of permeation. The result indicated that NEMGs changed to Emulgel suspension after 100 h, and the permeation rate decreased.

### 3.17. LZ Permeation/Day for 15 Days (ng/cm^2^ Versus Time in Hours)

**Figure 7:**
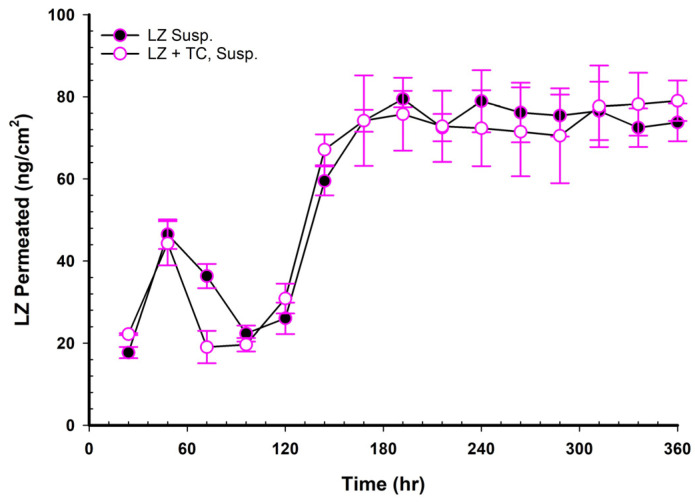
Plot of the daily permeated amount (ng/cm^2^) of LZ from suspensions up to 15 days.

**Figure 8:**
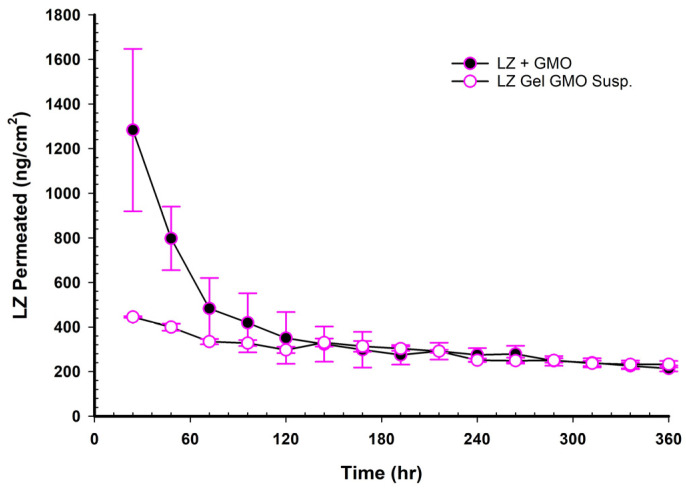
Plot of the daily permeated amount (ng/cm^2^) of LZ from GMO/NEMGs versus. GMO Emulgel suspension up to 15 days.

**Figure 9:**
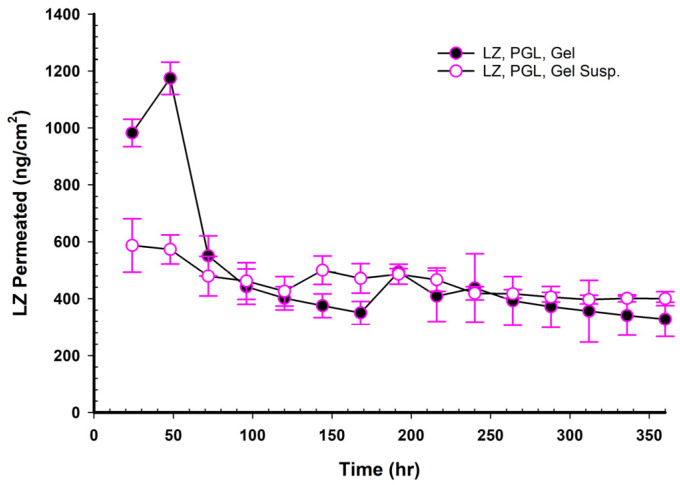
Plot of the daily permeated amount (ng/cm^2^) of LZ from GMO/PGL/NEMGs versus PGL Emulgel suspension up to 15 days.

**Figure 10:**
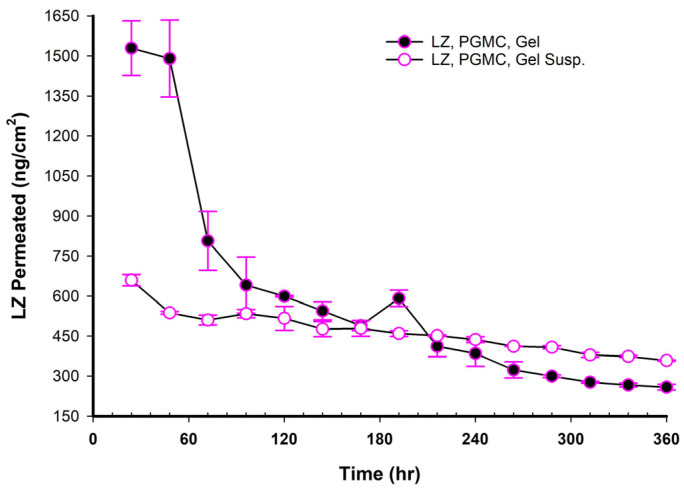
Plot of the daily permeated amount (ng/cm^2^) of LZ from GMO/PGMC/NEMGs versus PGMC Emulgel suspension up to 15 days.

**Figure 11:**
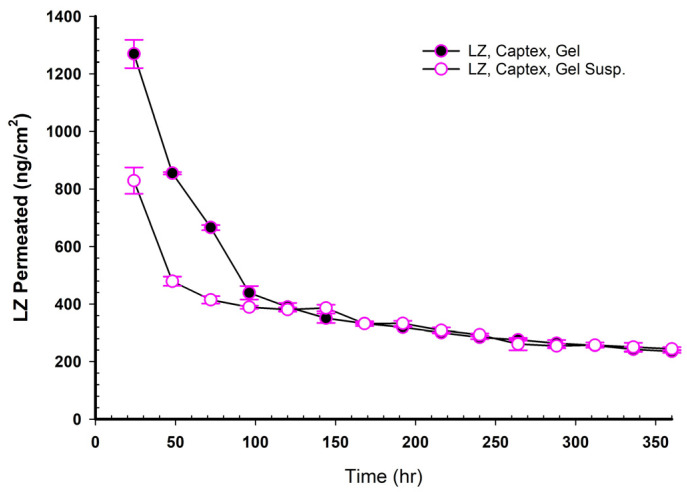
Plot of the daily permeated amount (ng/cm^2^) of LZ from GMO/Captex^®^/NEMGs versus Captex^®^ Emulgel suspension up to 15 days.

Figure 7, Figure 8, Figure 9, Figure 10 and Figure 11 illustrated the plots of the amount of LZ permeated per day versus time in hours over 15 days. Figure 7 presents two profiles of LZ suspensions in phosphate buffer at pH 6.8, one with and one without 20% TC. The two profiles were nearly superimposed, indicating that the 20% *v*/*v* TC has no significant effect on the permeation of LZ from the suspension in phosphate-buffer solution. After 150 h, the amount of LZ permeated remained relatively constant up to 15 days (360 h). This observation implied that the quantity of LZ dissolved from the suspended solids in the suspension replaced the amount of LZ penetrated, maintaining the thermodynamic activity of the solution at a steady level.

When NEMGs and their counter-Emulgel suspensions were plotted against time over a 15-day timeframe, as shown in Figure 8, Figure 9, Figure 10 and Figure 11, the amount of LZ permeated daily (24 h) by Emulgel suspensions was lower during the first 72 h than the amount permeated by NEMGs. After 100 h, the LZ penetration rates of Emulgel suspensions and NEMGs were almost equal, nearing a steady state. The components of NEMGs and Gel suspensions are shown in Table 9.

Table 9 shows that gel suspensions and NEMGs have almost the same percentage *w*/*w* of NEMG base. In contrast to Emulgel suspensions, NEMGs exhibited greater initial permeation during 72 h, especially for GMO/NEMG and GMO/PGMC NEMGs. This period implied that NEMGs functioned as nanoemulgels and changed gradually into an Emulgel suspension. According to a microscopical examination, LZ started to crystallize out of the NEMGs samples. The slow solvent dragging of TC, PG, Polysorbate 80, and PE over the membrane towards the receptor medium may have been the direct cause of this transformation; however, more research is required to confirm this occurrence. After 100 h, the behavior of NEMGs was comparable to that of Emulgel suspensions, with a steep negative slope and a nearly constant LZ permeation rate. The diffusion was predominantly in suspensions for NEMGs and Emulgel suspensions.

### 3.18. In Vivo PK Studies

#### HPLC Analysis & Validation

The 1–1000 ng/mL calibration curve showed a 0.9997 linear regression. The selectivity was checked as blank plasma was subjected to the extraction procedure, spiked with an internal standard, and did not show interfering peaks in the LZ retention time. It was due to the high selectivity of the fluorescence detector. According to the ICH Guideline M10 for bioanalytical method validation, the method’s sensitivity showed that the LOQ was 1 ng/mL and the LOD was 0.33 ng/mL. As previously reported [30,31], LZ’s average recovery was almost 100%, matching the published value of 96.94 ± 2.66%. This suggested that during extraction, a tiny amount was lost. Because they showed a higher in vitro permeation rate than other formulations, the nanoemulgel formulation and its suspension gel systems (PGMC and PGMC suspension) were chosen for in vivo PK studies.

**Figure 12:**
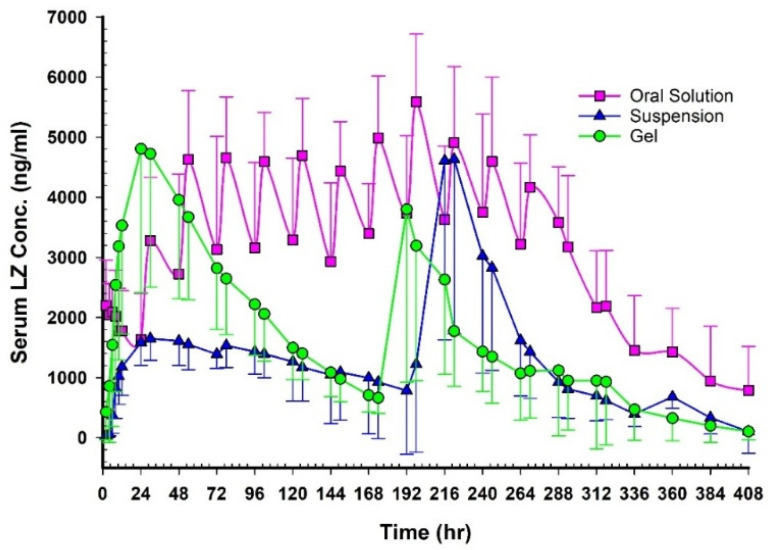
LZ’s mean plasma concentration-time profiles following oral solution, NEMG, and Emulgel suspension transdermal administrations.

Figure 12 shows plasma concentration–time profiles after oral, NEMG, and Emulgel administration. Table 10 and Table 11 summarize the key pharmacokinetic parameters. Following multiple oral administrations, the concentration-time profile of LZ at a dosage of 1 mg per day over 12 days revealed eleven absorption peaks. In contrast, double peaks were observed following transdermal applications using GMO/PGMC NEMG and GMO/PGMC Emulgel suspension, indicating two distinct phases of permeation. The appearance of dual peaks in the plasma concentration profile may be attributed to time-dependent changes in the formulation. The initial peak likely corresponds to the diffusion of LZ from the freshly prepared formulation, which contained higher concentrations of cosolvents and penetration enhancers. As these components gradually migrated and their concentrations decreased within the formulation, LZ diffusion slowed, giving rise to the second plasma peak. This phenomenon was supported by the in vitro permeation studies (Figure 8, Figure 9, Figure 10 and Figure 11), where the first 100 h exhibited the highest daily LZ diffusion rates, closely aligning with the initial plasma concentration peak. The secondary peak may thus be linked to the subsequent reduction in LZ diffusion observed beyond 100 h, as depicted in Figure 8, Figure 9, Figure 10 and Figure 11.

The relative bioavailability of LZ following transdermal application of the two patches, compared to oral solution administration, was approximately 57% for GMO/PGMC NEMG and 41% for GMO/PGMC Emulgel suspension, as reported in Table 11. The surface area of each patch measured 2.55 cm^2^, which appeared insufficient to facilitate the complete permeation of LZ from NEMGs and gel suspension patches.

The oral solution exhibited a higher C_max_ and bioavailability than both gels; however, the AUC was only significant (*p* < 0.05). This may be attributed to the fact that not all 12 mg of LZ could permeate from the topical gels, with a limited surface area of 2.55 cm^2^. Over the initial 96 h, there were no significant differences in C_max_ and AUC between the oral solution and the GMO/PGMC NEMG (*p* < 0.05). This finding indicated that the nanoemulgel structure permeated effectively, comparable to the oral solution of LZ in ethanol. As presented in Table 11, the geometric mean ratio of LZ (at a 90% Confidence Interval (CI), GMO/PGMC NEMG) indicated a C_max_ comparable to that of the oral solution, measured at 0.89 (range: 0.7–1.12). The permeation surface area was recorded at 2.55 cm^2^, which, if increased, could facilitate the attainment of bioequivalence.

Acknowledged skin permeability and drug metabolism differences between rats and humans should be carefully considered in transdermal drug delivery research, particularly during preclinical evaluation. While rat skin generally shows higher permeability and faster drug elimination than human skin, in vitro experiments can still provide reliable predictions of human skin permeability. Moreover, integrating pharmacokinetic parameters can improve the accuracy of interspecies comparisons [55].

Furthermore, there were no discernible variations between the two emulgels regarding C_max_ and AUC (*p* > 0.05). However, the C_max_ and AUC_0–174_ for NEMGs were substantially greater than the equivalent AUC_0–174_ for the Emulgel suspension (*p* < 0.05), while the AUC_174–360_ did not show a significant difference (*p* > 0.05). As previously mentioned, the dominant nanoemulgel structure of GMO/PGMC NEMG is responsible for the first phase. The suspension gel dominated the permeation in the second phase for both emulgels, which led to no significant changes in the C_max_ and AUC of LZ (*p* > 0.05). This observation is consistent with the in vitro permeation data for GMO/PGMC NEMG and GMO/PGMC Emulgel suspension, as shown in Figure 10.

### 3.19. Shelf-Life Stability of LZ Nanoemulgels

After almost 2 years of storage at room temperature, no significant physical and chemical changes were observed for any of the LZ/GMO/PGMC, NEMG, and LZ/GMO/PGMC Emulgel suspension (Assay: 100.4 ± 2.23% for the first sample and 99.1 ± 1.60% for the second sample). Crystal growth was not observed. Table 12 summarizes the stability data of LZ–PGMC NEMGs.

### 3.20. Accelerated Stability Studies of LZ Nanoemulgels and Emulgel Suspensions

**Figure 13:**
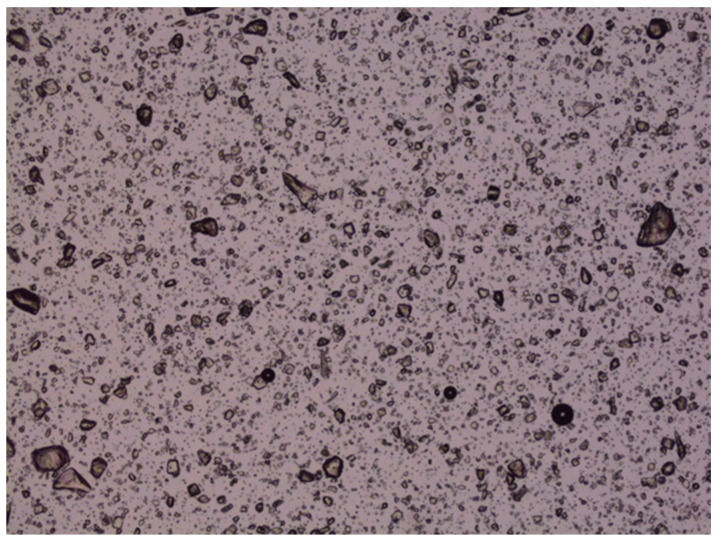
Photomicrograph of LZ in GMO/PGMC Emulgel suspension before storage at 50 °C.

**Figure 14:**
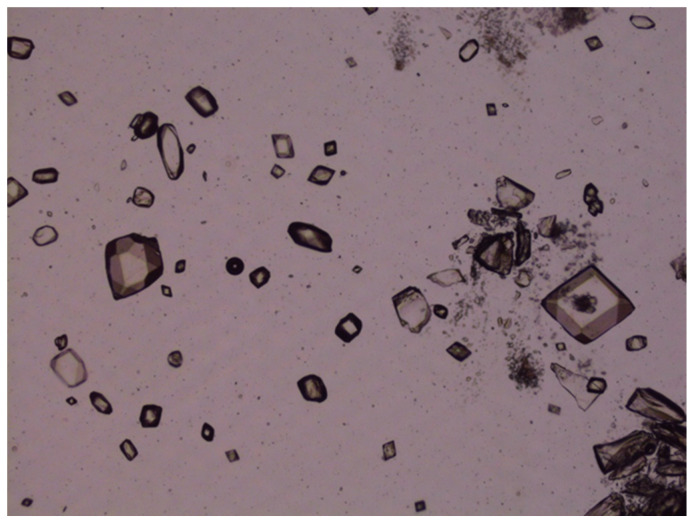
Photomicrograph of LZ in GMO/PGMC Emulgel suspension after storage at 50 °C for one month, showing crystal growth of LZ.

The accelerated stability studies conducted at 50 °C over 40 days demonstrated that the chemical stability of various LZ nanoemulgels and emulgel suspensions, which included different penetration enhancers, was preserved (Assay: 100.6–102.4% with nearly 100% peak purity). However, crystal growth was noted in LZ emulgels that contained suspended particles of LZ, as illustrated in Figure 13 and Figure 14. This occurrence was anticipated due to the presence of cosolvents and surfactants, which contributed to the thermodynamic instability of the emulgel suspensions at the elevated temperature of 50 °C.

In summary, the results of the preliminary stability investigations (both accelerated and shelf-life) indicated that LZ nanoemulgels and emulgel suspensions were expected to remain stable for a commendable period of two years. Nevertheless, further stability studies are necessary. These should include long-term assessments under varying temperature conditions (25 °C and 30 °C), humidity levels (60%, 65%, and 75% RH), and accelerated stability testing at 40 °C and 75% RH over six months.

## 4. Conclusions

This study developed and characterized non-aqueous LZ/NEMG formulations for transdermal drug delivery. The preparation process for the non-aqueous NEMGs was straightforward, and the formation of the NEMG occurred spontaneously, resulting in a clear, thick gel. The optimized GMO/PGMC-based NEMG exhibited superior permeation, a biphasic pharmacokinetic behavior, and a good stability profile. In vivo evaluations demonstrated sustained plasma concentrations and a bioavailability of 57% relative to the oral solution for the 2.55 cm^2^ patch. This suggested that transdermal nanoemulgels could be a clinically viable alternative to oral administration by increasing the surface area. The LZ/GMO/PGMC Emulgel suspension, conversely, demonstrated reduced permeability and lower relative bioavailability due to the loss of the emulgel’s nanostructure, an essential factor in enhancing the permeation of LZ.

By facilitating controlled releases, minimizing systemic side effects, and improving patient adherence, LZ nanoemulgels showed considerable promise for enhancing therapeutic outcomes in breast cancer management. Future research should concentrate on scaling production, assessing long-term stability under ICH guidelines, and conducting clinical evaluations in humans to ascertain their translational potential.

## Figures and Tables

**Table 1:** LZ’s solubility (µg/mL, mean ± SD) in various solvents and cosolvents at 32 °C and 37 °C.

Solvent	Solubility at 32 °C	Solubility at 37 °C
Water	47.2 ± 0.27	48.2 ± 2.62
Phosphate buffer pH 6.8	45.6 ± 0.32	---
Propylene glycol	748.1 ± 31.72	1721.8 ± 241.66
Isopropyl alcohol	1476.5 ± 66.09	2543.2 ± 257.98
Oleic acid	222.1 ± 13.74	254.1 ± 24.10
Peceol^®^	88.2 ± 14.67	202.4 ± 32.54
Labrasol^®^	2281.6 ± 228.17	4376.0 ± 417.34
Transcutol^®^	30,209.9 ± 4355.35	82,132.6 ± 2562.81
PEG-400	4561.4 ± 816.18	25,478.3 ± 3609.83
Ethanol	4567.1 ± 154.41	5634.7 ± 628.51
Ethyl Oleate	213.8 ± 26.27	337.5 ± 64.54
Isopropyl myristate	279.0 ± 29.25	374.7 ± 39.10

**Table 2:** LZ solutions’ stability in various pharmaceutical additives kept at 50 °C for 1 month (mean ± SD).

	Concentration (µg/mL)				
Solvent	Initial Conc (C_0_)	(1 Week)	(2 Weeks)	(1 Month)	% Decomp.
Water	47.2 ± 0.27	49.0 ± 1.12	48.9 ± 1.09	48.6 ± 0.53	None
PHB pH 6.8	45.6 ± 0.32	48.8 ± 1.55	49.8 ± 1.33	49.7 ± 1.24	None
Propylene glycol	748.1 ± 31.72	759.4 ± 28.03	776.2 ± 47.83	774.1 ± 55.28	None
Isopropyl alcohol	1476.5 ± 66.09	1555.6 ± 54.84	1562.8 ± 24.43	1516.5 ± 63.09	None
Oleic acid	222.1 ± 13.74	216.2 ± 15.25	212.2 ± 15.38	194.0 ± 19.05	4.5
GMO/Abitec	102.4 ± 14.67	----	-----	100.2 ± 12.51	2.2
Labrasol^®^	2281.6 ± 228.17	2612.2 ± 265.68	2446.8 ± 354.19	2307.3 ± 445.44	None
Transcutol^®^	30,209.9± 4355.35	29,320.0 ± 5761.60	31,246.4 ± 3113.18	29,544.2 ± 4443.6	2.2
PEG-400	4561.4 ± 816.18	4047.1 ± 510.07	3652.6 ± 235.32	3100.1 ± 451.83	32.1
Ethanol	4567.1 ± 154.41	5478.8 ± 932.66	6148.1 ± 998.5	13,504.8 ± 11,956.3	None
Isopropyl myristate	279.0 ± 29.25	293.7 ± 19.55	276.5 ± 34.65	265.3 ± 31.32	4.9

**Table 3:** Nanoemulgel components’ percentages and their Appearances.

Formula	LZ	SG	GMO	TC	PG	GMO Component *	Gel Appearance
Placebo	0.00	19.98	12.72	34.85	32.54	Campul^®^	Clear Gel
F01	1.31	17.87	14.37	39.53	26.92	Campul^®^	Clear Gel
F02	1.29	20.37	12.63	34.26	31.45	Campul^®^	Clear Gel
F03	1.32	18.16	14.46	39.20	26.87	Campul^®^	Clear Gel
F04	1.51	18.16	14.46	39.00	26.87	Campul^®^	Clear Gel
F05	1.30	18.34	14.75	39.17	26.44	Myverol^®^	Clear Gel with Lumps
F06	1.51	12.11	9.73	45.58	31.07	Myverol^®^	Clear Gel
F07	1.35	13.31	11.09	40.95	33.3	Peceol^®^	Turbid
F08	1.52	19.49	0.00	44.96	34.03	None	Almost Clear Gel
F09	1.34	20.02	9.62	38.84	30.18	Campul^®^/Peceol^®^, 1:1	Clear Gel
F10	1.31	18.42	15.08	38.63	26.56	Myverol^®^/Campul^®^/ Peceol^®^, 2:1:1	Clear Gel

* Peceol^®^ (Glyceryl monooleate of 40% content); Campul^®^ (GMO of 60% content); Myverol^®^ (GMO of 90% content).

**Table 4:** Nanoemulgel Formulations Containing Penetration Enhancers (PE): Components as % *w*/*w* and Gel Appearance.

	GEL				
Component	LZ	LZ/GMO	LZ/GMO/PGL	LZ/GMO/PGMC	LZ/GMO/Captex
LZ	1.52	1.34	1.15	1.58	1.58
SG	19.49	20.02	16.80	16.93	17.90
Campul^®^	0.00	9.62	13.00	11.30	14.10
PG	34.03	38.83	32.48	26.60	26.50
TC	44.96	30.18	30.90	38.55	35.00
PGL	0.00	0.00	5.72	0.00	0.00
PGMC	0.00	0.00	0.00	5.06	0.00
Captex^®^	0.00	0.00	0.00	0.00	5.00
Appearance	Clear, thick	Clear, thick	Clear, thick	Clear, thick	Clear, thick

**Table 5:** Permeation parameters for LZ from phosphate buffer suspensions with or without TC. Each value represents mean ± SD (*n* = 4).

	Flux, (µg/cm^2^·h)	T_lag_, h (±SD)	Permeation Coef. × 10^−5^ (cm/h)	SD × 10^−5^
LZ Suspension in PHB (pH = 6.8)	2.42	81.4 (3.761)	2.4	0.12
LZ Suspension in PHB (pH = 6.8) with 20% TC	2.43	77.4 (7.329)	2.4	0.22

**Table 6:** Permeation parameters for LZ from NEMGs. Each value represents mean ± SD (*n* = 4).

NEMGs Composition	Flux, (µg/cm^2^·h)	T_lag_, h (±SD)	Permeation Coef. × 10^−5^ (cm/h)	SD × 10^−5^
LZ, GMO	16.67 (0.868)	7.2 (0.372)	111.1	5.8
LZ. NO GMO	5.69 (0.962)	40.3 (6.928)	37.9	6.7
LZ, GMO/PGL	20.56 (0.921)	25.6 (1.116)	137.1	6.3
LZ, GMO/PGMC	24.75 (1.229)	8.8 (0.426)	165.0	7.8
LZ, GMO/Captex^®^	18.00 (0.310)	9.1 (0.160)	120.0	2.3

**Table 7:** Permeation parameters for LZ from Gel Suspensions. Each value represents mean ± SD (*n* = 4).

Emulgel Suspension	Flux, (µg/cm^2^·h)	T_lag_, h (±SD)	Permeation Coef. × 10^−5^ (cm/h)	SD × 10^−5^
LZ, GMO	12.48 (0.427)	26.9 (0.903)	19.4	0.62
LZ, GMO/PGL	19.13 (0.278)	34 (0.521)	29.8	0.46
LZ, GMO/PGMC	19.41 (0.221)	30.4 (0.338)	30.2	0.34
LZ, GMO/Captex^®^	15.03 (0.417)	14.9 (0.327)	23.4	0.75

**Table 8:** The permeation enhancement factor (PEF) of permeation of LZ in NEMGs or Emulgel suspensions (TDDS) relative to LZ suspension in phosphate buffer.

TDDS (LZ in NEMGs and LZ in Gel Suspensions).	PEF
LZ suspension in phosphate buffer of pH 6.8	1.0
LZ suspension in phosphate buffer of pH 6.8 containing 20% TC	1.0
LZ NEMG (NO GMO)	15.8
LZ/GMO, NEMG	46.3
LZ/GMO/PGL, NEMG	57.1
LZ/GMO/PGMC, NEMG	68.8
LZ/GMO/Captex^®^, NEMG	50
LZ/GMO, Emulgel suspension	8.0
LZ/PGL, Emulgel suspension	12.4
LZ/PGMC, Emulgel suspension	12.6
LZ/Captex^®^, Emulgel suspension	9.8

**Table 9:** The nominal % *w*/*w* of each component in NEMGs and Emulgel Suspensions.

Component	% *w*/*w* in NEMGs	% *w*/*w* in Emulgel Suspension
Dissolved LZ	1.5	1.43
Suspended LZ	0	5
NEMG Base	98.50	93.56

**Table 10:** Summary of the pharmacokinetic parameters of LZ.

Formulation	N	AUC_0–t_ (ng·h/mL)	AUC_inf_ (ng·h/mL)	C_max_ (ng/mL)	t_max_ (h)	t_0.5_ (h)
Oral Solution	12	1.13 × 10^6^ (32.0)	1.17 × 10^6^ (39.3)	6040 (17.4)	174 (54.0, 246.0)	37.3 (7.27)
Emulgel Suspension	12	472,000 (57.3)	474,000 (57.6)	3900 (104)	216 (48.0, 360.0)	18.9 (5.2)
Nanoemulgel	12	654,000 (32.5)	660,000 (31.6)	5370(46.1)	27.0(24.0, 216.0)	26.4 (5.68)

**Table 11:** Summary of the geometric mean ratio for the pharmacokinetic parameters of Emulgel suspensions and LZ NEMG in relation to the oral solution of LZ.

Treatment	PK Parameter	Ratio of Geometric LZ Means (90% CI)
Nanoemulgel/Oral Solution	AUC_0_**_–_**_t_, ng·h/mL	0.58 (0.46–0.72)
Nanoemulgel/Oral Solution	AUC_0_**_–_**_Inf_, ng·h/mL	0.57 (0.43–0.74)
Nanoemulgel/Oral Solution	C_max_, ng/mL	0.89 (0.7–1.12)
Emulgel Suspension/Oral Solution	AUC_0_**_–_**_t_, ng·h/mL	0.42 (0.31–0.57)
Emulgel Suspension/Oral Solution	AUC_0_**_–_**_Inf_, ng·h/mL	0.41 (0.28–0.59)
Emulgel Suspension/Oral Solution	C_max_, ng/mL	0.65 (0.42–0.99)

**Table 12:** Long-term room temperature stability data of LZ-PGMC Nanoemulgel.

LZ-PGMC Nanoemulgel (1.58% *w*/*w*)	Mean, % LZ	SD
Assay, initial concentration	99.1	2.11
Assay after 17 mo. and 5 days at RT	99.0	1.08
Assay after 25 mo. and 10 days at RT	100.2	1.03

## Data Availability

Data are contained within the article.

## Abbreviations

The following abbreviations were used in this manuscript:

ACUC	Animal Care and Use Committee
AI	Aromatase Inhibitor
ANOVA	Analysis of Variance
AUC	Area Under the Curve
AUC_0–t_	Area Under the Plasma Concentration–Time Curve to Time t
AUC_0–∞_	Area Under the Plasma Concentration–Time Curve Extrapolated to Infinity
CI	Confidence Interval
Cmax	Maximum Plasma Concentration
DDS	Drug Delivery System
GMO	Glyceryl Monooleate
HCl	Hydrochloric Acid
HPLC	High-Performance Liquid Chromatography
ICH	International Council for Harmonisation
LOD	Limit of Detection
LOQ	Limit of Quantification
LZ	Letrozole
NaOH	Sodium Hydroxide
NEMGs	Non-aqueous Nanoemulgels
PCL	Polycaprolactone
PE	Penetration Enhancer
PEF	Permeation Enhancement Factor
PEG	Polyethylene Glycol
PG	Propylene Glycol
PGMC	Propylene Glycol Monocaprylate
PGL	Propylene Glycol Laurate
PHB	Phosphate Buffer
PK	Pharmacokinetics
QC	Quality Control
RH	Relative Humidity
RSD	Relative Standard Deviation
SD	Standard Deviation
SG	Sepineo P600^®^ (Acrylamide/Sodium Acryloyldimethyl Taurate Copolymer) Gel
TC	Transcutol^®^ (Diethylene Glycol Monoethyl Ether)
TD	Tadalafil
TDDS	Transdermal Drug Delivery System
Tmax	Time to Reach Maximum Concentration
UPLC	Ultra-Performance Liquid Chromatography
UV	Ultraviolet
WHO	World Health Organization

## Appendix A

**Table A1:** Accuracy/Recovery Results using Nanoemulgel Base.

Accuracy/Recovery Test/Nanoemulgel Base	Amount Recovered	Average Assay %	RSD%
Spiked Sample, Level 80%	79.6	99.5	0.2
Spiked Sample, Level 100%	99.2	99.2	0.6
Spiked Sample, Level 120%	119.64	99.7	1.1

**Table A2:** Intraday and Interday Precisions.

		Intra-Day Precision	Inter-Day Precision
Sample	Conc. (µg/mL)	RSD (%)	RSD (%)
1	5	0.4	1.7
2	25	0.82	1.98
3	75	0.59	1.71

**Table A3:** Forced degradation studies of LZ.

Sample	% Recovered	% Decomposed
Control	100	0
0.1 M HCl	98.53	1.47
0.1 M NaOH	28.4	71.6
5% H_2_O_2_	98.54	1.46
Thermal	99.87	0.13
UV-Radiation	100	0

## References

[1] Wilkinson L., Gathani T. (2022). Understanding breast cancer as a global health concern. Br. J. Radiol..

[2] International Agency for Research on Cancer (IARC), WHO (2025). Breast Cancer.

[3] Kim J., Harper A., McCormack V., Sung H., Houssami N., Morgan E., Mutebi M., Garvey G., Soerjomataram I., Fidler-Benaoudia M.M. (2025). Global patterns and trends in breast cancer incidence and mortality across 185 countries. Nat. Med..

[4] Bekes I., Huober J. (2023). Extended Adjuvant Endocrine Therapy in Early Breast Cancer Patients-Review and Perspectives. Cancers.

[5] Alemrayat B., Elrayess M.A., Alany R.G., Elhissi A., Younes H.M. (2018). Preparation and optimization of monodisperse polymeric microparticles using modified vibrating orifice aerosol generator for controlled delivery of letrozole in breast cancer therapy. Drug Dev. Ind. Pharm..

[6] Buzdar A.U., Robertson J.F., Eiermann W., Nabholtz J.M. (2002). An overview of the pharmacology and pharmacokinetics of the newer generation aromatase inhibitors anastrozole, letrozole, and exemestane. Cancer.

[7] Mukherjee A.G., Wanjari U.R., Nagarajan D., KK V., Chakraborty R., Renu K., Dey A., Vellingiri B., Gopalakrishnan A.V. (2022). Letrozole: Pharmacology, toxicity and potential therapeutic effects. Life Sci..

[8] Pfister C.U., Martoni A., Zamagni C., Lelli G., De Braud F., Souppart C., Duval M., Hornberger U. (2001). Effect of age and single versus multiple dose pharmacokinetics of letrozole (Femara) in breast cancer patients. Biopharm. Drug Dispos..

[9] National Center for Biotechnology Information (2025). PubChem Compound Summary for CID 3902, Letrozole. https://pubchem.ncbi.nlm.nih.gov/compound/Letrozole.

[10] Li L., Fang L., Xu X., Liu Y., Sun Y., He Z. (2010). Formulation and biopharmaceutical evaluation of a transdermal patch containing letrozole. Biopharm. Drug Dispos..

[11] Choudhury H., Gorain B., Pandey M., Chatterjee L.A., Sengupta P., Das A., Molugulu N., Kesharwani P. (2017). Recent Update on Nanoemulgel as Topical Drug Delivery System. J. Pharm. Sci..

[12] Pople P.V., Singh K.K. (2006). Development and evaluation of topical formulation containing solid lipid nanoparticles of vitamin A. AAPS PharmSciTech.

[13] Ansari A., Verma M., Majhi S. (2025). Nanoemulgel: A Comprehensive Review of Formulation Strategies, Characterization, Patents and Applications. Micro Nanosyst..

[14] Gao L., Gao L., Huang S., Sun L., Li M., Shen C., Chen Y., Tan R., Chen Y., Zhan C. (2025). Nanoemulsion-based transdermal delivery of third-generation steroidal and non-steroidal aromatase inhibitors in preclinical models. Cell Prolif..

[15] Sallam A.A., Younes H.M. (2021). Transdermal Non-Aqueous Nanoemulgels for Systemic Delivery of Aromatase Inhibitors. U.S. Patent.

[16] Khan S.U., Ullah M., Saeed S., Saleh E.A.M., Kassem A.F., Arbi F.M., Wahab A., Rehman M., ur Rehman K., Khan D. (2024). Nanotherapeutic approaches for transdermal drug delivery systems and their biomedical applications. Eur. Polym. J..

[17] Cevc G., Vierl U. (2010). Nanotechnology and the transdermal route: A state of the art review and critical appraisal. J. Control. Release.

[18] Lal D.K., Kumar B., Saeedan A.S., Ansari M.N. (2023). An Overview of Nanoemulgels for Bioavailability Enhancement in Inflammatory Conditions via Topical Delivery. Pharmaceutics.

[19] Ramadon D., McCrudden M.T.C., Courtenay A.J., Donnelly R.F. (2022). Enhancement strategies for transdermal drug delivery systems: Current trends and applications. Drug Deliv. Transl. Res..

[20] Karande P., Mitragotri S. (2009). Enhancement of transdermal drug delivery via synergistic action of chemicals. Biochim. Biophys. Acta (BBA)—Biomembr..

[21] Zhang X., Wu C.C., Jiang H., Zhao J.F., Pan Z.J., Zheng Y. (2025). The Role of Thickening Agent Proportions in Optimizing Nanoemulsion Gel for Dermatophytosis Treatment. Int. J. Nanomed..

[22] Mou D., Chen H., Du D., Mao C., Wan J., Xu H., Yang X. (2008). Hydrogel-thickened nanoemulsion system for topical delivery of lipophilic drugs. Int. J. Pharm..

[23] Donthi M.R., Munnangi S.R., Krishna K.V., Saha R.N., Singhvi G., Dubey S.K. (2023). Nanoemulgel: A Novel Nano Carrier as a Tool for Topical Drug Delivery. Pharmaceutics.

[24] Kadukkattil Ramanunny A., Singh S.K., Wadhwa S., Gulati M., Kapoor B., Khursheed R., Kuppusamy G., Dua K., Dureja H., Chellappan D.K. (2022). Overcoming hydrolytic degradation challenges in topical delivery: Non-aqueous nanoemulsions. Expert Opin. Drug Deliv..

[25] Sun R., Xia N., Xia Q. (2020). Non-aqueous nanoemulsions as a new strategy for topical application of astaxanthin. J. Dispers. Sci. Technol..

[26] Bhagat V., Rachh P. (2021). Formulation and development of lipid based non-aqueous nano emulsion for selected NSAID. J. Adv. Sci. Res..

[27] Borman P., Elder D. (2017). Q2(R1) Validation of Analytical Procedures. ICH Quality Guidelines.

[28] Assaf S.M., Ghanem A.M., Alhaj S.a.A., Khalil E.A., Sallam A.A. (2022). Formulation and Evaluation of Eudragit^®^ RL Polymeric Double Layer Films for Prolonged-Release Transdermal Delivery of Tamsulosin Hydrochloride. AAPS PharmSciTech.

[29] Zhang Y., Huo M., Zhou J., Zou A., Li W., Yao C., Xie S. (2010). DDSolver: An add-in program for modeling and comparison of drug dissolution profiles. AAPS J..

[30] Acharjya S.K., Bhattamisra S.K., Muddana B.R., Bera R.V., Panda P., Panda B.P., Mishra G. (2012). Development of a high-performance liquid chromatographic method for determination of letrozole in wistar rat serum and its application in pharmacokinetic studies. Sci. Pharm..

[31] Zarghi A., Foroutan S.M., Shafaati A., Khoddam A. (2007). HPLC Determination of Letrozole in Plasma Using Fluorescence Detection: Application to Pharmacokinetic Studies. Chromatographia.

[32] Younes H. Development of RP-UPLC method for the assay and stability evaluation of letrozole in pharmaceutical emulgels. Proceedings of the Qatar Foundation Annual Research Forum.

[33] Patel R.B., Patel M.R., Thakore S.D., Patel B.G., Grumezescu A.M. (2017). Chapter 17—Nanoemulsion as a Valuable Nanostructure Platform for Pharmaceutical Drug Delivery. Nano- and Microscale Drug Delivery Systems.

[34] Shah M.H., Paradkar A. (2007). Effect of HLB of additives on the properties and drug release from the glyceryl monooleate matrices. Eur. J. Pharm. Biopharm..

[35] Dante M.C.L., Borgheti-Cardoso L.N., Fantini M.C.A., Praça F.S.G., Medina W.S.G., Pierre M.B.R., Lara M.G. (2018). Liquid Crystalline Systems Based on Glyceryl Monooleate and Penetration Enhancers for Skin Delivery of Celecoxib: Characterization, In Vitro Drug Release, and In Vivo Studies. J. Pharm. Sci..

[36] Haque T., Talukder M.M.U. (2018). Chemical Enhancer: A Simplistic Way to Modulate Barrier Function of the Stratum Corneum. Adv. Pharm. Bull..

[37] Gavinet B., Sigurani S., Garcia C., Roso A. (2024). Alternatives to Conventional Topical Dosage Forms for Targeted Skin Penetration of Diclofenac Sodium. Int. J. Mol. Sci..

[38] Uchida T., Kadhum W.R., Kanai S., Todo H., Oshizaka T., Sugibayashi K. (2015). Prediction of skin permeation by chemical compounds using the artificial membrane, Strat-M™. Eur. J. Pharm. Sci..

[39] Haq A., Dorrani M., Goodyear B., Joshi V., Michniak-Kohn B. (2018). Membrane properties for permeability testing: Skin versus synthetic membranes. Int. J. Pharm..

[40] Assaf S.M., Sallam A.S.A., Ghanem A.M. (2019). Design and evaluation of transdermal delivery system containing tamsulosin hydrochloride. J. Drug Deliv. Sci. Technol..

[41] Kong M., Chen X.G., Kweon D.K., Park H.J. (2011). Investigations on skin permeation of hyaluronic acid based nanoemulsion as transdermal carrier. Carbohydr. Polym..

[42] Pereira G.R., Collett J.H., Garcia S.B., Thomazini J.A., Bentley M.V.L.B. (2002). Glycerol monooleate/solvents systems for progesterone transdermal delivery: In vitro permeation and microscopic studies. Rev. Bras. Cienc. Farm..

[43] Kadhum W.R., See G.L., Alhijjaj M., Kadhim M.M., Arce F.J., Al-Janabi A.S., Al-Rashidi R.R., Khadom A.A. (2022). Evaluation of the Skin Permeation-Enhancing Abilities of Newly Developed Water-Soluble Self-Assembled Liquid Crystal Formulations Based on Hexosomes. Crystals.

[44] Mistry J., Notman R. (2024). Mechanisms of the Drug Penetration Enhancer Propylene Glycol Interacting with Skin Lipid Membranes. J. Phys. Chem. B.

[45] Musakhanian J., Osborne D.W., Rodier J.-D. (2024). Skin Penetration and Permeation Properties of Transcutol^®^ in Complex Formulations. AAPS PharmSciTech.

[46] Abd E., Namjoshi S., Mohammed Y.H., Roberts M.S., Grice J.E. (2016). Synergistic Skin Penetration Enhancer and Nanoemulsion Formulations Promote the Human Epidermal Permeation of Caffeine and Naproxen. J. Pharm. Sci..

[47] Rajan R., Vasudevan D. (2012). Effect of Permeation Enhancers on the penetration Mechanism of transfersomal gel of ketoconazole. J. Adv. Pharm. Technol. Res..

[48] Preeti, Sambhakar S., Malik R., Bhatia S., Al Harrasi A., Rani C., Saharan R., Kumar S., Geeta, Sehrawat R. (2023). Nanoemulsion: An Emerging Novel Technology for Improving the Bioavailability of Drugs. Scientifica.

[49] Ghasemiyeh P., Mohammadi-Samani S. (2020). Potential of Nanoparticles as Permeation Enhancers and Targeted Delivery Options for Skin: Advantages and Disadvantages. Drug Des. Dev. Ther..

[50] Shakeel F., Baboota S., Ahuja A., Ali J., Shafiq S. (2008). Skin permeation mechanism and bioavailability enhancement of celecoxib from transdermally applied nanoemulsion. J. Nanobiotechnol..

[51] Karve T., Dandekar A., Agrahari V., Melissa Peet M., Banga A.K., Doncel G.F. (2024). Long-acting transdermal drug delivery formulations: Current developments and innovative pharmaceutical approaches. Adv. Drug Deliv. Rev..

[52] Mohammed D., Hirata K., Hadgraft J., Lane M.E. (2014). Influence of skin penetration enhancers on skin barrier function and skin protease activity. Eur. J. Pharm. Sci..

[53] Morin G.M.D. (2019). Synergising Excipients to Boost Skin Delivery. ONdrugDelivery Mag..

[54] Keservani R.K., Bandopadhyay S., Bandyopadhyay N., Sharma A.K., Tekade R.K. (2020). Chapter 4—Design and fabrication of transdermal/skin drug-delivery system. Drug Delivery Systems.

[55] Todo H. (2017). Transdermal Permeation of Drugs in Various Animal Species. Pharmaceutics.

